# The morphology of patella changed significantly after soft tissue correction for children with recurrent patella dislocation

**DOI:** 10.1186/s12891-020-03846-6

**Published:** 2020-12-10

**Authors:** Jinghui Niu, Qi Qi, Kuo Hao, Wei Lin, Kang Piao, Fei Wang

**Affiliations:** 1 Department of joint surgery, The third hospital of Hebei Medical University, Shijiazhaung City, Hebei Province China; 2 Department of Cardiology, The third hospital of Hebei Medical University, Shijiazhaung City, Hebei Province China

**Keywords:** Patella, Patellar morphology, Patellar dislocation, Children

## Abstract

**Background:**

Although morphological improvement of femoral trochlea has been investigated in children with patellar dislocation after surgery, whether the patellar shape changed under the same condition is still unknown. The purpose of the study was to investigate the changes of patellar morphology in transverse plane following surgical correction of recurrent patellar dislocation in children.

**Methods:**

A total of 22 patients with a mean age of 9.9 years (7 to 12) were included. All had unilateral recurrent patellar dislocation. The knees with recurrent patellar dislocation were treated with medial patellar retinaculum plasty and defined as the affected side. The contralateral knees with no symptom of patellar instability were considered as the unaffected side. All patients were treated between October 2014 and August 2018. Axial CT (Computed Tomography) scans were undertaken in all patients to assess the patella morphological characteristics on a particular axial image preoperatively and at the final follow-up.

**Results:**

There were not significant differences about patellar transverse diameter, thickness and Wiberg angle between affected side and unaffected side before surgery and at the last follow-up respectively (The mean follow-up period: 28.0 ± 3.3 months). However, before surgery, the Wiberg-index in the affected side (0.74 ± 0.06) was significantly different from that in the unaffected side (0.64 ± 0.04). At the last follow-up, the Wiberg-index in the affected side (0.67 ± 0.05) and the unaffected side (0.65 ± 0.04) were not significantly different. Also, in the affected side, the Wiberg-index at the last follow-up was significantly lower than that before surgery (*P* < 0.05). The Wiberg-index in the unaffected side was not significantly different before surgery and at the last follow-up.

**Conclusion:**

The patellar morphology can change significantly after surgical procedures in children with patellar recurrent dislocation whose epiphysis is not closed.

## Background

The patella is the largest sesamoid bone in the body and articulates with femoral trochlear [1]. In the articular part of the patella, a median crest can be found, defining a medial and a lateral facet [2]. The femoral trochlea consists of the lateral and medial facet of the femoral sulcus which allow patella to remain centred in the trochlea during normal knee movement [3]. The patella lies superior to the trochlear cartilage at full knee extension and begins to articulate with femoral trochlea as the knee flexes to 30°. The contact area between patella and femoral trochlea is approximately 2.1 cm^2^ at 30° of knee flexion and increases to approximately 5.5 cm^2^ at 90° of knee flexion [4, 5].

If the patella locates out of the femoral trochlea, the patellar dislocation occurs. Patellar dislocation is a common disease among children and teenagers, and the incidence is over 30 per 100,000. Up to 71% of these patients may develop recurrent patellar instability, and 20% go on to suffer patellofemoral arthritis [6–9].

There are four possible factors for patellar instability: trochlear morphologic characteristics, limb geometries in the axial plane, patellar height ratios, and ligamentous stabilizers [10]. Among them, the patellar dysplasia with a longer lateral facet and femoral trochlear dysplasia with a higher sulcus angle have been regarded as predisposing factors for the patellar dislocation [11–13].

On the other hand, a series of studies on the topic of if the patellar dislocation and surgical correction for treating patellar dislocation have significant effects on the patellofemoral joint development have been published. Li and Wang found femoral trochlear dysplasia after patellar dislocation in growing rabbits [14, 15]. Niu found the axial shape and articular surface of the patella became more flattened after patella instability in growing rabbits [16]. These studies indicated that patellofemoral joint dysplasia could be caused by patellar dislocation. Also, some clinical studies focused on the topic. Benoit et al. [17] reported that children with recurrent patellar dislocation had a statistically significant improvement in the femoral sulcus angle after patellar stabilization procedure. Fu et al. [18] found that the femoral trochlear morphology can be improved with lower trochlear groove angle and higher trochlear groove depth by early surgical correction in children with recurrent patellar dislocation associated with femoral trochlear dysplasia.

Although patella articulates with femoral trochlea, to our best knowledge, there is no study focusing on the morphological alteration of patella after surgical procedures in children with recurrent patellar dislocation. This is the first study focusing on the topic. Based on the previous studies, we hypothesized that patellar morphology improves significantly after surgical procedures in children with recurrent patella dislocation.

## Methods

This is a retrospective study. The patients who met the inclusions were taken into account. The study had ethical approval of ethics committee of local hospital (No. G2016–020-1) and all patients gave informed consents.

The inclusion criteria were: unilateral recurrent patella dislocation (more than there dislocations or dislocation still occurs after three-month conservative treatments) and the contralateral knee with no symptoms of patella instability; the patients were under 12 years old and with open growth plate (the ossification of patella completes by age 13–16 years) [19]; patella with type II and III as defined by Wiberg in the dislocated knee [20]; medial patellar retinacular plasty combined with lateral retinaculum release or not was performed unilaterally.

The exclusion criteria were: cases with closed physes; concomitant cruciate ligament or collateral ligament injury; rheumatoid arthritis or osteonecrosis with cartilage damage of greater than grade II [21]; Other surgical procedures such as medial patellofemoral ligament reconstruction, tibial tubercle transfer and femoral trochlear osteotomy; Patellar redislocation in the follow-up.

At last, a total of 22 patients (age 7–12) who were treated between October 2014 and August 2018 were enrolled. Ten patients had traumatic patellar dislocation. And the mean time from injury to surgery was 2.2 ± 0.7 months. The period from pre-op CT scans and surgery was 0.9 ± 0.6 months. The knees with recurrent dislocation were treated with medial patellar retinacular plasty and were defined as the affected side. The contralateral knees were considered as the unaffected side.

### Surgical techniques

The surgeries were completed by one experienced senior surgeon. Before performing medial patellar retinacular plasty, arthoscopic assessment was performed and chondral lesions and concomitant pathology were treated. A force-directed medial shift of the patella was performed and the movement was less than one quarter the width of the patella indicated overtension of the lateral retinaculum. The lateral retinacular release was undertaken in these patients [22].

The plasty procedures had been illustrated by Fu and Ji [18, 23]. The procedures vary from the position of the injury. For the injury of the medial patellar retinaculum near the patellar attachment or midportion, the junction of the vastus medialis obliquus and the medial patellar retinaculum were incised transversely. At 30 degrees of knee flexion, the medial patellar retinaculum was pulled proximally and sutured to the medial proximal border of the patella with PDS-I whipstitch, then the vastus medialis oblique muscle fibre was pulled distally and sutured to the medial distal border of the patella over the retinaculum.

**Fig. 1:**
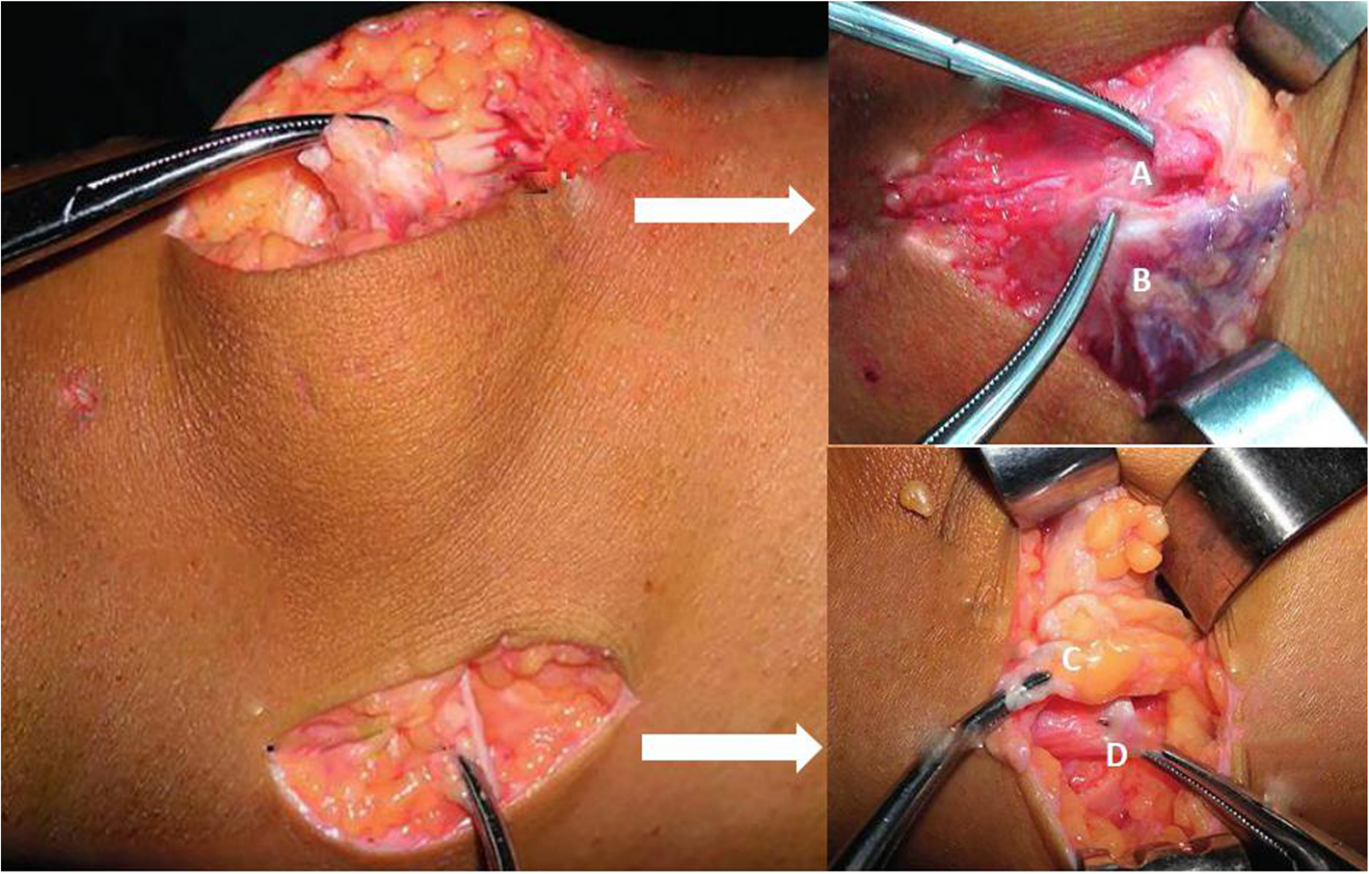
The surgical procedures for the patient with medial retinaculum injury at patellar site and femoral site. **A** The isolated medial retinaculum was pulled proximally to the medial upper border of the patella. **B** The vastus medialis was pulled distally over the medial retinaculum. **C** The proximal part of the medial retinaculum was pulled distally. **D** The distal part of the medial retinaculum was pulled proximally

For the injury of the medial patellar retinaculum near the femoral attachment, a transverse incision was made at the injured inferior border of medial patellar retinaculum and the two parts were clamped separately. The distal part was pulled to the proximal border of the adductor tubercle while the proximal part was pulled to the distal border of the adductor. Then the two parts were sutured with PDS-I whipstitch. Figure 1 is for the patients with injury both near patellar site and femoral attachment of patellar retinaculum.

### Postoperative management

On the second day after surgery, leg raising and quadriceps isometric strength exercises were performed. One week after surgery, a gradual range of motion was initiated with a hinged brace. Four weeks after operation, knee flexion with 90 degrees was achieved. At 6 weeks after surgery, the brace was removed and full weight-bearing was allowed without a crutch. Three months postoperatively, the patients are able to perform normal daily activities. Contact sports were permitted 6 months after the surgery.

### Assessments

Knee function was evaluated using Kujala score before surgery and at the follow-up [24]. Physical examination was assessed using the apprehension test for lateral stability of the patella. Knee joint CT scans in the axial plane were performed to measure patella tilt angle, patella congruence angle, trochlea sulcus angle and TT-TG distance preoperatively and at the final follow-up on both sides. In addition, patella tilt angle had be evaluated preoperatively and postoperatively on both sides.

**Fig. 2:**
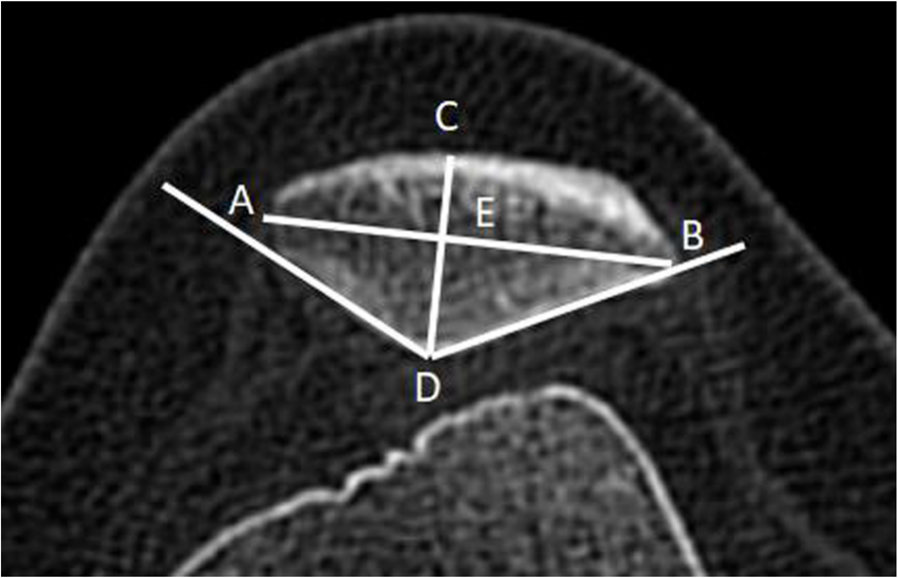
The measurements of the patella. Line **AB**: the transverse diameter of the patella. Line **CD**: the line is vertical to Line **AB** and represents for the thickness of the patella. Point E: the insertion of Line **AB** and Line **CD**. Angle **D**: Wiberg-angle

For patellar morphology measurements (Fig. 2), the slice image with the widest diameter of the patella was used for the measuring in the axial plane. The line between the most medial point A and the most lateral point B of the patella was defined as the transverse diameter (Line AB) [25]. The posterior patellar point farthest from the Line AB was defined as point D. The thickness of the patella was measured by the length of line CD which is vertical to the Line AB. The insertion between line CD and line AB was defined as point E. The Wiberg angle (∠D) was measured, and Wiberg index (length of BE/length of AB) was calculated as description by Fucentese et al. [11]. The measurements were performed in RadiAnt-DICOM software (Medixant Ltd., Poznan, Poland), which provides an accuracy of 0.01 mm for length and 0.1° for angle. Intra-observer and interobserver consistency were performed.

### Statistical analysis

Before the investigation, the sample size was estimated using Wiberg-index as the primary variable. On the basis of previous study [11], the standard deviation was assumed at 0.06 in both affected and unaffected sides. An estimated difference between the groups was set as 0.06. A power calculation was performed by G-power Software (3.1.9.6) with a confidence level of 95% (α = 0.05) and power (1-β) of 80%. An estimated sample size of 17 knees per group was yielded.

Statistical analysis was performed using the SPSS version 21.0 (SPSS, IL, USA). The normality and homogeneity of the data were tested by the Kolmogorov–Smirnov test and Levene’s test respectively. All numerical variables showed a normal distribution and equal variance. Student’s *t* test was used to test the difference of the numerical variables. A *P* value < 0.05 was determined as statistically significant. The results are expressed as mean ± standard deviation.

To determine the intra-observer variation, the observer A repeated the measurements 2 weeks after first observing. To determine the inter-observer variation, the measurements were measured by observer A, observer B and observer C. Intra-observer and interobserver consistency were analysed using the intra-class correlation coefficient (ICC). ICC > 0.75 was regarded as excellent, ICC 0.40–0.75 was fair to good, and ICC < 0.40 was poor.

## Results

Eight males and 14 females were included in the study. Ten patients suffered from traumatic patellar dislocation. The average age at the surgery was 9.9 ± 1.4 years old. The follow-up period was 28.0 ± 3.3 months. No patellar redislocation case was found in the follow-up. All the patients had nagtive apprehension test results in the follow-up. The height, weight, Kujala score before surgery and at the last follow-up were showed at Table 1.

**Table 1:** Characteristics of the patients before surgery and at the follow-up

	Before Surgery	At the last follow-up
Height (mm)	145.0 ± 12.0	157.0 ± 10.4
Weight (Kg)	40.0 ± 6.2	47.7 ± 6.8
Kujala Score	59.0 ± 7.6	87.6 ± 5.7

In the affected side (Table 2), the patellar tilt angle (28.4 ± 7.1°) and congruence angle (22.8 ± 6.3°) before surgery decreased significantly at the last follow-up (patellar tilt angle: 10.7 ± 2.3°; congruence angle: 8.4 ± 2.6°). The sulcus angle in the affected side (148.2 ± 7.2°) was significantly different from that at the last follow-up (144.1 ± 5.4°), which was consistent with the study by Fu [18].

**Table 2:** Measurements of the patellofemoral joint before surgery and at last follow-up

Measurement	Group	Before surgery	At last follow-up	*P* Value
PTA(°)	affected side	28.4 ± 7.1	10.7 ± 2.3	< 0.01
	unaffected side	9.2 ± 2.5	9.4 ± 2.1	0.746
SA(°)	affected side	148.2 ± 7.2	144.1 ± 5.4	0.038
	unaffected side	137.7 ± 5.4	139.3 ± 5.9	0.357
TT-TG (mm)	affected side	17.91 ± 3.67	17.54 ± 3.21	0.725
	unaffected side	12.20 ± 2.04	13.41 ± 1.50	0.031
CA(°)	affected side	22.8 ± 6.3	8.4 ± 2.6	< 0.01
	unaffected side	4.8 ± 3.4	4.4 ± 3.1	0.647

*PTA* patellar tilt angle, *SA* sulcus angle, *TT-TG* tibial tuberosity trochlear groove distance, *CA* congruence angle

By Table 3, there were not significant differences about transverse diameter, thickness and Wiberg angle between affected side and unaffected side before surgery and at the last follow-up respectively. However, the Wiberg-index in the affected side (0.74 ± 0.06) was significantly different from that in the unaffected side (0.64 ± 0.04) before surgery. At the last follow-up, the Wiberg-index in the affected side (0.67 ± 0.05) and the unaffected side (0.65 ± 0.04) were not significantly different. Also, in the affected side, the Wiberg-index at the last follow-up was significantly lower than that before surgery (*P*<0.05). The Wiberg-index in the unaffected side was not significantly different before surgery and at the last follow-up.

**Table 3:** Measurements of the patella before surgery and at the last follow-up

Measurement	Affected side	Unaffected side	*P* Value
Diameter (mm) before surgery	3.34 ± 0.37	3.27 ± 0.39	0.53
Diameter (mm) at the last follow-up	3.75 ± 0.30	3.72 ± 0.37	0.74
Thickness (mm) before surgery	1.63 ± 0.32	1.71 ± 0.36	0.42
Thickness (mm) at the last follow-up	1.84 ± 0.33	1.94 ± 0.41	0.36
Wiberg-angle (°) before surgery	140.2 ± 14.3	141.8 ± 12.0	0.69
Wiberg-angle (°)at the last follow-up	141.6 ± 13.1	139.2 ± 10.2	0.51
	*P* = 0.75	*P* = 0.44	
Wiberg-index before surgery	0.74 ± 0.06	0.64 ± 0.04	< 0.01
Wiberg-index at the last follow-up	0.67 ± 0.05	0.65 ± 0.04	0.10
	*P* < 0.01	*P* = 0.64	

**Fig. 3:**
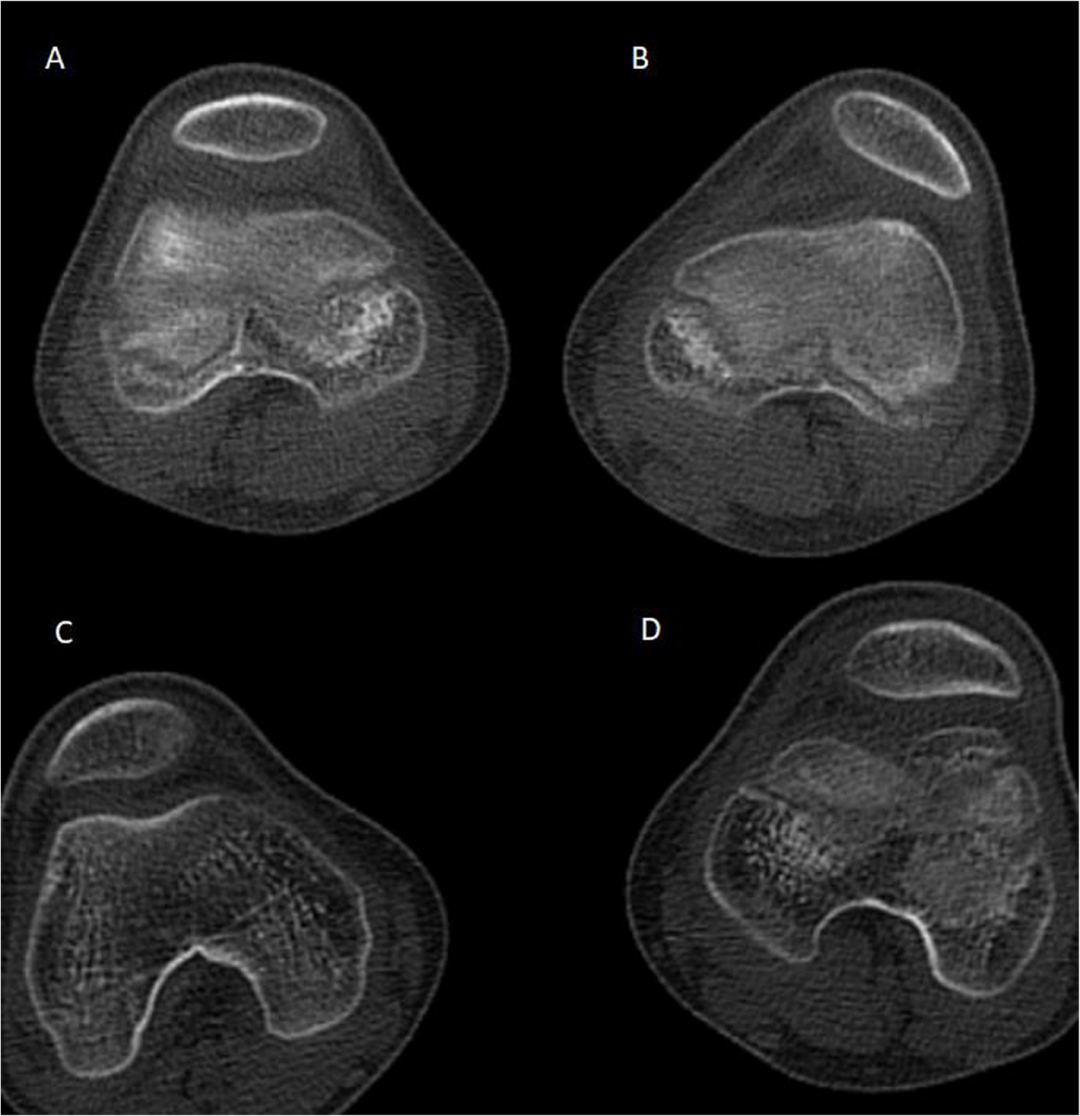
The axial CT of the patella before surgery and at the last follow-up in one patient. Patella **A** is in the unaffected side and Wiberg-index is 0.61. Patella **B** is in the affected side and the Wiberg-index is 0.78. Patella **C** develops from patella **A** at the last follow-up and the Wiberg-index is 0.64. Patella **D** develops from patella **B** at the last follow-up and the Wiberg-index is 0.69

The Intra-observer consistency and interobserver consistency are showed in Table 4. The axial CT of one patient was shown in Fig. 3.

**Table 4:** Inter- and Intraobserver Reliability of the Different Measurements

Intraclass Correlation Coefficient (95% CI)			
Measurements		Before surgery	Last follow-up
Diameter	Intraobserver Reliability	0.986 (0.974–0.992)	0.935 (0.625–0.978)
	Interobserver Reliability	0.924 (0.874–0.956)	0.893 (0.829–0.937)
Thickness	Intraobserver Reliability	0.982 (0.967–0.990)	0.981 (0.966–0.990)
	Interobserver Reliability	0.931 (0.887–0.960)	0.949 (0.901–0.973)
Wiberg-angle	Intraobserver Reliability	0.978 (0.942–0.990)	0.966 (0.935–0.981)
	Interobserver Reliability	0.900 (0.841–0.940)	0.913 (0.863–0.949)
Wiberg-index	Intraobserver Reliability	0.912 (0.754–0.961)	0.899 (0.535–0.963)
	Interobserver Reliability	0.852 (0.771–0.911)	0.827 (0.729–0.896)

## Discussion

The most important finding of the present study was before the surgery, the Wiberg-index in the affected side was significantly higher than that of the unaffected side. At the last follow-up after surgical corrections, the Wiberg-index between the affected side and the unaffected side were not significantly different, thus leading a more developed medial facet of the patella in the affected side.

For skeletally immature patients, the femoral attachment of the medial patellofemoral ligament is close to the medial femoral physis. And the midpoint of the medial patellofemoral ligament is at, slightly above or slightly below the physis [26]. The procedures with medial retinaculum plasty have been used to treat recurrent patella dislocation in skeletally immature pediatric patients as it does not threaten open physes.

The average PTA in the affected side before surgery was 28.4° and decreased to 10.7° at the last follow-up. A significant association between patellar shape and patellar tilt: the more the patella is tilted, the more the patella is dysplastic was found by Panni [12]. The patella with a high patellar tilt angle increases stress on the lateral facet and decreases stress on the medial facet. This condition may arise during growth and cause a hypoplastic medial facet and a more developed lateral facet according to Wolff’s law. So the Wiberg-index in affected side was significantly higher than that of the unaffected side in this study.

After the surgical correction, the patellar dislocation and patella tilt had been corrected. Thus the medial facet of the patella had a more normal contact area and pressure with femoral trochlea. This had been proved by Yamada [27]. He found for normal knees, the contact area on the femoral trochlea extended medially with increasing knee flexion. The patellas contacted with the medial facet of the trochlea by 30° in all knees. After that, this band-like area of contact moved downwards on both the medial and lateral facets of the femoral trochlea with increasing knee flexion. Also in the study [27], for 14 patients with patella instability, only two patellas were in contact with the medial facet and these two patients were successfully treated by conservative treatments. In the present study, because of the normal condition of the stress and contact area in the patellofemoral joint after surgery, the patellar morphology improves by developing medial facet more than before. And at the final follow-up, the Wiberg-index between the affected side and the unaffected side are not significantly different.

When comes with the development of the patellofemoral joint, the patella and femoral trochlear should be observed and analyzed as a whole [28]. Type B and type C patella were more likely to have a flatter, more shallow trochlear groove (*P* < 0.05) [29]. A smaller medial patellar facet and an increased Wiberg-index were usually found in the knees with trochlear dysplasia [30]. It is possible that the articulation between the patella and femoral trochlea plays a role in the shaping of each other during early skeletal development. The poor engagement between them can lead to trochlear and/or patellar dysplasia and the correction of the poor engagement should improve it.

The results in the study also inspire us the exploration for the influence factors of the patellofemoral development. Only minor changes in the anatomical development of the femoral trochlea from newborn to age 6 years were found [31]. The surface morphology of the knee is determined very early in utero and the fetal patellar morphology is comparable with that of adults [32–34]. In the present study, the Wiberg-index and Wiberg-angle in the unaffected side are almost same before surgery and at the last follow-up, which is consistent with the previous studies. On the other hand, patellar and trochlear dysplasia may be induced by experimental patellar dislocation in animal models [14–16]. The present study and the study by Fu [18] showed significant morphological alteration of patella and femoral trochlea after surgical correction in the children with recurrent patella dislocation. So the development of the patella and femoral trochlea was thought to be effected on both congenital factors and acquired factors, especially the engagement between patella and femoral trochlea.

Combined with the previous study [14–16, 18], the normal patella position with femoral trochlea is important for the patellofemoral development. Patellar and trochlear dysplasia may be induced by experimental patellar dislocation in animal models [14, 15]. And effective treatments for patellar dislocation which retain the normal contact of patellofemoral joints can achieve morphological remodeling (more developed medial patellar facet and more deepened femoral trochlea) in the follow-up, which is protective against the further dislocation and can be beneficial for the skeletally immature patients.

There are some limitations about the study. First, the number of the patients (22 patients) is low. More patients met the inclusion criteria should be analyzed in the future. But power analysis showed 17 knees is necessary for experimental or unaffected side. Second, CT can be used to describe the osseous contour which cannot be matched with the corresponding surface, which is covered with cartilage when viewed by MRI. The CT was chosen because it is cheaper and faster than MRI.

## Conclusion

The Wiberg-index in the affected side decreased significantly after soft tissue correction in children with recurrent patellar dislocation, which indicated a more developed medial facet of patella. This indicated that the patellar morphology could change after surgical procedures in skeletal immature patients with patellar recurrent dislocation.

## Data Availability

The datasets used during the current study are available from the corresponding author on reasonable request.
